# Provision of non-invasive coronary and carotid vascular imaging results on changes in diet and physical activity in asymptomatic adults: A scoping review

**DOI:** 10.3389/fnut.2022.946378

**Published:** 2022-10-28

**Authors:** Simone Radavelli-Bagatini, Abadi K. Gebre, Mary A. Kennedy, Marc Sim, Lauren C. Blekkenhorst, Catherine P. Bondonno, Ben Jackson, James Dimmock, Markus P. Schlaich, Jonathan M. Hodgson, Joshua R. Lewis

**Affiliations:** ^1^Nutrition and Health Innovation Research Institute, School of Medical and Health Sciences, Edith Cowan University, Perth, WA, Australia; ^2^Medical School, The University of Western Australia, Perth, WA, Australia; ^3^School of Human Sciences (Exercise and Sport Science), The University of Western Australia, Perth, WA, Australia; ^4^Telethon Kids Institute, Perth, WA, Australia; ^5^Department of Psychology, College of Healthcare Sciences, James Cook University, Townsville, QLD, Australia; ^6^Dobney Hypertension Centre, Royal Perth Hospital Unit/Medical School, The University of Western Australia, Perth, WA, Australia; ^7^Department of Cardiology, Royal Perth Hospital, Perth, WA, Australia; ^8^Department of Nephrology, Royal Perth Hospital, Perth, WA, Australia; ^9^School of Public Health, Sydney Medical School, Children's Hospital at Westmead, Centre for Kidney Research, The University of Sydney, Sydney, NSW, Australia

**Keywords:** coronary artery calcification (CAC), carotid ultrasound (CUS), vascular imagining, cardiovascular disease (CVD), diet and physical activity change

## Abstract

**Background:**

Although a healthy diet and physical activity have been shown to prevent or delay cardiovascular disease (CVD) hospitalizations and deaths, most adults do not meet current guidelines. Provision of coronary artery calcification (CAC) and carotid ultrasound (CUS) imaging results may motivate beneficial lifestyle changes. We scoped the existing literature for studies providing non-invasive vascular imaging results and reporting diet, physical activity, and/or anthropometric measures to identify knowledge gaps and opportunities for further research.

**Methods:**

A systematic search was performed across three electronic databases, in line with PRISMA ScR guidelines and Arksey and O'Malley's scoping review framework.

**Results:**

Twenty studies (thirteen observational and seven randomized controlled trials) examining the impact of provision of CAC/CUS imaging results on diet and/or physical activity behaviors were included. Nearly half the studies did not clearly state whether participants received dietary and physical activity advice along with vascular imaging results, and these were secondary outcomes in most studies, with data assessment and reporting being inconsistent.

**Conclusion:**

Well-designed clinical trials with consistent and clear messaging based on detailed subjective and objective measures of diet and physical activity are needed to determine whether this approach may stimulate long-term dietary and physical activity change.

## Introduction

Cardiovascular disease (CVD) accounts for almost 1 in 3 deaths globally, with the majority (85%) attributable to either ischemic heart disease or cerebrovascular disease (1). Most survivors have substantially impaired quality of life due to ongoing functional deficits (2, 3). Suboptimal lifestyle behaviors are the leading causes of CVD globally (4), and most CVD-related events could be prevented or substantially delayed by improving diet, increasing physical activity, and ceasing smoking (1). Even modest sustained lifestyle changes can reduce CVD risk (5). Despite evidence showing that high consumption of fruit and vegetables (FV) can lead to an estimated 20% lower risk of CVD, compared to low FV intake (a 5% lower risk for each additional serving) (6), only 51 and 8% of Australians meet the minimum recommended 2 serves of fruit (≥300 g/d) and 5 serves of vegetables (≥375 g/d) daily (7, 8), respectively. Moreover, only 15% of adults age 18–64 years meet the recommended amount of physical activity (9) each week. Clearly, currently policies and strategies to increase FV intake to recommended amounts have not been successful. New strategies are warranted to further encourage a healthier diet and lifestyle, aiming at improving heart health, and provision of vascular health may further encourage these changes. High quality evidence from large randomized controlled trials (RCTs) and meta-analyses has shown that provision of vascular imaging can increase behavior change resulting in improved medication adherence in the long term (10–12), highlighting its potential utility as a promising approach to elicit diet and physical activity change. This is because people are more likely to make healthy changes if they perceive they are at risk of developing a disease and that the condition can lead to serious consequences (13).

Vascular health is commonly assessed in the coronary arteries (coronary artery calcification [CAC]) or in the carotid arteries (carotid ultrasound [CUS]). CAC testing has been increasingly used in clinical practice to screen asymptomatic patients for advanced atherosclerosis (14, 15) and to identify asymptomatic individuals at higher risk of future cardiovascular events (16). CUS is used to assess common carotid artery intimal medial thickness (cIMT) and focal carotid atherosclerotic plaques, as measures of carotid atherosclerosis (17). Detection of CAC using computed tomography (CT) and ultrasound, has been shown to strongly predict future cardiovascular events (16) among asymptomatic individuals. It has been proposed that such imaging techniques could provide superior insight as a marker of CVD risk, beyond conventional risk factors (18).

Although the impact of provision of vascular health imaging on medication adherence is well-known, the effects on diet and physical activity behaviors are less certain due to the lack of well-designed clinical trials with a focus on those outcomes. A systematic review and meta-analysis including six studies with a total of 11,256 participants on the impact of provision of CAC results on medication initiation and continuation, as well as on preventive lifestyle changes, observed improvements in the use of aspirin (OR [95%CI]: 2.6 [1.8–3.8], lipid-lowering medication: 2.9 [1.9–4.4], hypertension medication: 1.9 [1.6–2.3] and continuation of lipid-lowering medication: 2.3 [1.6–3.3]) (19). In addition, participants with abnormal CAC scans significantly improved their diet and exercise (OR [95%CI]: 1.9 [1.5–2.5] and 1.8 [1.4–2.4], respectively), compared to those with absence of CAC (19). However, no studies have mapped the literature to understand key features of these studies and highlight the areas of focus for improving diet and physical activity as part of provision of vascular health imaging.

The aim of this scoping review was to map the literature to understand the evidence to date and identify opportunities to implement diet and physical activity interventions to support changes in these areas. We sought to summarize the literature to identify the nature of study participants, understand how messages were conveyed to participants and which recommendations were provided, as well as to highlight which tools were used to measure the outcomes of interest, and the duration of follow-up period. The outcomes of this study may allow us to identify gaps in the literature to guide the design of high-quality RCTs.

## Methods

Our study was guided by Arksey and O'Malley (20) scoping review framework and included five stages: (1) the research question was identified; (2) relevant studies were flagged; (3) suitable studies were selected; (4) data was charted and; (5) relevant information was collated, summarized and described. The PRISMA Extension for Scoping Reviews (PRISMA-ScR) checklist was also used to guide this review (21).

### Research questions

A scoping review question was established with the view of broadly scoping the evidence in the literature that suggests provision of non-invasive carotid or coronary vascular imaging can promote healthy diet and physical activity change, with best practices being not clear:

*By scoping the literature to identify studies in asymptomatic adults that provided non-invasive carotid or coronary vascular imaging and measured diet and/or physical activity, can we determine which approaches may lead to healthy long-term changes in diet and physical activity, in whom and why?*

### Eligibility criteria

The search for studies focused on the effects of knowledge of vascular health across the adult lifespan on changes in diet and physical activity, and anthropometric measures (as a result of changes in diet and physical activity). Full text original articles available in English with no restriction on year of publication were included. The inclusion criteria for studies were: (1) adult men and women without prior diagnosis of CVD; (2) carotid or coronary vascular imagining results provided to participants; (3) studies with information on changes in diet and/or physical activity and/or anthropometric measures after provision of vascular imaging results. We used these inclusion criteria to reflect an approach to primary prevention using vascular imaging results.

### Search strategy and study selection

A comprehensive online literature search of scientific papers listed on Medline, Embase, and CINAHL was undertaken from database inception until January 24th, 2022. A hand search of references and gray literature sources were also performed (e.g., Google Scholar), to ensure that all relevant articles were included. Table 1 shows the search terms included in this review. Although the term “behavior change” is broad and may include behaviors not related to diet and physical activity (psychological behavior changes, for example), this was included in our search to identify any publications that may have included any of our outcomes of interest and avoid missing relevant studies. To ensure that variations of all keywords were retrieved, keywords and combinations of keywords were used, as well as the wildcard symbol (^*^). No limits or filters were used for year of publication. Only articles published in English and including adults (age ≥18 years) were considered. Only data related to pre-established outcomes of interest were extracted.

**Table 1:** Study search terms.

**Exposures**
‘vascular calci*' OR ‘coronary calci*' OR ‘coronary artery disease' OR ‘carotid stenosis' OR ‘carotid arter*' OR ‘atherosclerotic plaque*' OR ‘arter* plaque'
**AND (assessments)**
‘comp* tomography' OR angiography OR ‘X-ray computed' OR ultraso* OR CT
**AND (broad key words)**
‘life style change' OR ‘lifestyle change' OR ‘lifestyle behav*' OR ‘behav* change' OR ‘behav* modification' OR ‘health behav*' OR ‘motivation to change' OR ‘risk factor* change' OR ‘risk factor* modification'

Search terms using individual and combinations of keywords, as well as the wildcard symbol (*), with no limits or filters for year of publication. Only articles published in English and including adults (age ≥18 years) were considered.

**Figure 1:**
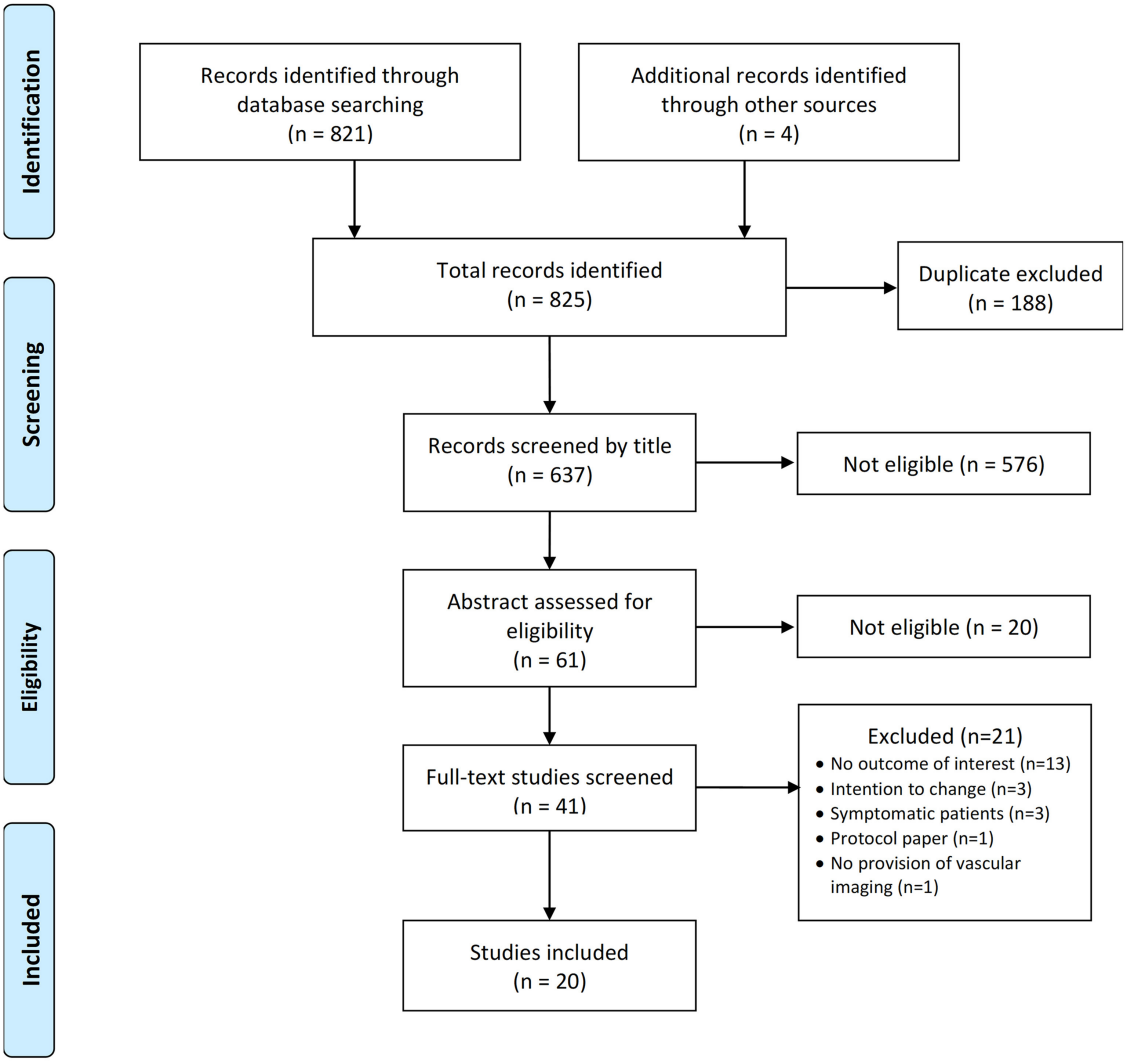
Flow diagram for the scoping review process.

A total of 821 publications were retrieved using the search terms, and 4 other articles were retrieved by hand search. Figure 1 shows the PRISMA flow diagram. All references were imported into EndNote software and screened for duplicates (*n* = 188). Screening of the remaining 637 studies was conducted in the following order: (i) article title; (ii) abstract; and (iii) full text. Two authors (SRB and AKG) screened all articles for specific outcomes of interest. The following words were used to identify the outcomes of interest for this review: diet, exercise, physical activity, exercise, nutrition, weight, BMI, body mass index, body mass, body composition, waist circumference. Discrepancies between the screening authors were resolved through discussions with a senior author (JRL) until consensus was reached. A total of 20 studies were eligible and therefore included in this scoping review (Figure 1).

### Data charting and reporting

Data from each of the selected records were presented in a narrative format and sorted into tables in chronological order (oldest to newest publications). Pertinent data were retrieved from eligible articles including authorship, publication year, and country; population details (sample size, age, sex); methods and intervention details; outcomes of interest (for RCTs); follow-up period; and significant findings for the outcomes of interest, for both observational (Table 2) and RCT (Table 3) studies. The outcomes of interest found in the records included: overall diet composition, vegetable intake, fiber intake, fat intake, salt intake, consumption of processed foods, exercise/physical activity and anthropometric measures which included body weight and/or body mass index (BMI), and waist circumference.

**Table 2:** Summary of observational studies on provision of CAC and CUS results and changes in diet, physical activity and anthropometric measures.

**Author, year, country**	**Sample details (size, age, sex, type)**	**Methods**	**CAC or CUS classification**	**Findings based on the outcomes of interest**	**Follow-up period**
**Coronary artery calcification (CAC)**					
Wong et al. (22) US	Seven hundred and three asymptomatic adults with no CVD, 59% men; 53.7 ± 9.9 y, self-referred, who had CAC scans performed at Harbor-UCLA Medical Center (1992–1994)	Provision of CAC results were not reported. Follow-up questionnaire mailed ~1–2 y after the initial CAC scan, assessing health behaviors and medication use, as well as information on hospitalization and whether a physician had been consulted. Information on counseling not reported	CAC assessed by EBT, with scores determined according to Agatston	Presence of calcium was associated with losing weight and decreasing dietary fat (*p* < 0.05). Exercise levels remained the same (*p* > 0.05)	1–2 years
Kalia et al. (23), US	Five hundred and five asymptomatic adults with no CVD, 82% men, 61 ± 10 y, referred by physician	CAC scan images shown to participants, with CAC seen as bright white spots in their coronary arteries. Atherosclerosis was classified as none, mild, moderate or severe. CAC was described as identifying underlying coronary atherosclerosis and a heart disease predictor. Information on diet and lifestyle (D&L) counseling not reported	CAC assessed by EBT, with scores determined according to Agatston as: none, mild, moderate or severe	Participants were divided into quartiles (Q) according to baseline CAC score (Q1: 0–30, Q2: 31–149, Q3: 151–526 and; Q4: ≥527). Those in Q1 were least likely to make changes to their diet and cardiovascular exercise. Dietary change increased from 41 to 64% across quartiles (*p* = 0.001)	3 ± 2 years
Sandwell et al. (24), US	Three hundred and sixty four asymptomatic adults with no CVD, 53.7% men, 68.7 y, 82% of all adult residents of Rancho Bernardo, a southern California community, invited to take part in a study of heart disease risk factors (1972–1974)	Written CAC scan results and a graph were mailed to all participants within a week. Those with scores >1,000 received a phone call. All participants were asked to share their results with their physician. Information on D&L counseling not reported	CAC assessed by EBT, with scores determined according to Agatston as: 0–10 = low risk, 11–400 = moderate >400 = high risk for CAD	Among participants with suboptimal lifestyle before the scan, those with higher risk were more likely to make dietary changes, particularly on intake of fat (*p* = 0.007), cholesterol (*p* = 0.030), and salt (*p* = 0.059) compared with those with lower risk. Body weight did not change	6 months
Orakzai et al. (25), US	Nine hundred and eighty asymptomatic adults, 78% men, 60 ± 8 y, referred by physician	Participants were allocated to 4 groups based on their CAC scores. Physicians or technologists discussed the CAC scans with participants. CAC was seen as bright white spots and defined as identifying underlying coronary atherosclerosis and a risk factor for heart disease. Atherosclerosis was classified as none, mild, moderate or severe. Those with presence of CAC received recommendations to change lifestyle	CAC assessed by EBT, with scores determined according to Agatston: CAC = 0, CAC = 1–99, CAC = 100–399, and CAC≥400	Participants with CAC = 0 had the least changes in diet and exercise (33% and 44%, respectively), with a gradual increase observed with higher CAC scores (diet: 1–99, 40%; 100–399, 58%; >400, 56%; exercise 1–99, 62%; 100–399, 63%; >400, 67%;); all *p* < 0.001	3 ± 2 years
Schwartz et al. (26), US	Five hundred and ten asymptomatic adults, 42.8% men, 64 ± 9.69 y, physician or self-referred as a supplement to their preventive health care	CAC images and scores provided to participants by a physician, and counseled for lifestyle and risk factor modification, including diet and exercise advice, and smoking cessation, based on their CAC scores. Participants were informed that CAC was an underlying coronary atherosclerosis and associated with heart disease	CAC assessed by EBT, with scores determined according to Agatston	Participants with greater CAC scores were more likely to report increasing exercise (OR = 1.34, *P* = 0.02), improving diet (OR = 1.40, *P* < 0.01), and reducing alcohol intake (OR = 1.46, *p* = 0.05)	6 ± 1 years
Johnson et al. (27), US	One hundred and seventy four high-risk adults (≥3 major risk factors), 62% men, 58 ± 7.5 y, referred by physicians or self-referral to a private radiology center	CAC results provided by a nurse via a phone call ~2–3 days after patients' scan. Results were mailed to participants, and they were encouraged to follow up with their physician. Information on D&L counseling not reported	CAC determined according to Agatston score: 0 = no risk/normal, 1–10 = very low risk, 11–100 = mild risk, 101–400 = moderate risk, and >400 = high risk for a stenotic lesion	The study observed improvements in self-reported diet and exercise (data not shown)	3 months
Kalia et al. (28), US	Two thousand six hundred and eight asymptomatic adults, 72% men, 58 ± 8 y, referred by physicians or self-referral to University-Affiliated Disease Prevention Center in California	CAC scan images shown to participants, and presence and severity of atherosclerosis discussed. A physician discussed the risk factors associated with CAC and provided recommendations on nutrition, exercise, and smoking cessation for risk reduction, based on CAC scores	CAC scores were determined according to Agatston	Participants with CAC = 0 had the least weight loss (19.8%), with a gradual increase in weight loss observed with higher CAC scores (1–99, 23.4%; 100–399, 30.8%; ≥400, 33.6%; *p* < 0.001 for trend)	4.1 ± 3.2 years
Schurink et al. (29), The Netherlands	Three hundred and eighteen asymptomatic sportsmen aged ≥45 years attending routine sports medical examination who underwent additional cardiac CT imaging	At least two (sports) cardiologists and one radiologist reviewed the abnormal findings on the scans.^#^ Provision of CAC results to participants not reported. Those with CAC scores 100–400 were provided with lifestyle recommendations and advised on statin treatment	CAC scores were determined according to Agatston	Approximately 23% of sportsmen improved their lifestyle, with the majority reporting changes to a healthier diet (46.8%) and weight loss (35.9%)	7–30 months
**Carotid ultrasound (CUS)**					
Rodondi N et al. (30), Switzerland	Thirty smokers (at least 30 cigarettes/d), 56.7% men, 51.8 ± 9.4 y, self-referred	CUS performed in all smokers, plus smoking cessation counseling and therapy, and an educational tutorial on atherosclerosis, provided by a trained physician. Smokers with plaques received two images of their plaques and a 6-min educational tutorial on their ultrasound results. Smokers without plaques received a similar 6-min tutorial modified accordingly and were informed that their cardiovascular risk depended on other CVD risk factors and smoking, despite the absence of plaques. Those with suboptimal lipid or glucose levels received advice for lifestyle modification	Carotid atherosclerosis assessed by CUS, and defined as a focal widening of more than 50% relative to adjacent segment	PA did not increase from BL to 2 months in the group with presence of plaques (BL median ([IQR]: 1,517 [914–2,255] vs. 2 months: 3,437 [2,306–5,505] MET. Min/w [*p* = 0.02]), whereas PA increased in the group without plaques (BL: 1,517 [914–2,255] vs. 2 months: 3,437 [2,306–5,505] MET x Min/w [*p* = 0.02]). Body weight remained unchanged in both groups	1 week, 2 weeks and 2 months
Johnson et al. (31), US^*^	Five hundred and twenty nine adults with ≥1 risk factors and no CVD, 56.7% men, 54 ± 7 y, referred by physicians to the University of Wisconsin Vascular Health Screening Program	CUS was performed to assess cIMT and to identify carotid plaques. Information on D&L counseling not reported	cIMT in the highest >75th percentile for age, sex, and race, or carotid plaque presence	Advanced atherosclerosis did not predict changes in diet, exercise frequency, or long-term health-related behavior change	1 year
Johnson et al. (32), US	Three hundred and fifty five asymptomatic adults with ≥1 risk factors, 42% men, 53.6 ± 7.9y from 5 community, non-academic, primary care medical practices, screened during routine office visits	Participants with abnormal CUS were shown images of their arteries and received standardized education about the relationship of abnormal CUS with CVD. A primary care professional provided lifestyle recommendations for CVD risk-reduction and if indicated, medication use was recommended	Carotid atherosclerosis defined as cIMT >75th percentile or carotid plaque presence	Although an increase in exercise frequency and weight loss was reported by participants (34 and 37%, respectively), these changes were not predicted by CUS results. Abnormal CUS discreetly predicted reduced intake of dietary sodium (OR = 1.45, *p* = 0.002) and increased intake of fiber (OR = 1.55, *p* = 0.022)	1 month
Hong et al. (33), Korea	Three hundred and forty seven asymptomatic hypertensive adults, 54.5% men, 61 ± 8 y, from 22 hospitals in Korea, screened during routine office visits	CUS performed to assess cIMT and to identify carotid plaques. Results were informed to participants and recommendations provided by a physician. Participants were informed about the association of CAD with CVD and that presence of plaque or increased cIMT indicated increased risk for heart attack, stroke, and death. Information on D&L counseling not reported	Carotid atherosclerosis defined as carotid plaque or cIMT ≥0.9 mm	BMI decreased at 6 months only in the negative CUS group (*delta* = −0.1 ± 0.4 kg/m2, *p* = 0.006). Waist circumference remained unchanged at 6 months for both groups	6 months
Jeong et al. (34), Korea	Seven hundred and ninety seven adults with T2D, 49.6% men, 60 ± 9.5 y, recruited from 24 hospitals in Korea	CUS was performed to assess cIMT and to identify carotid plaques. Information on D&L counseling not reported	Carotid atherosclerosis defined as carotid plaque or cIMT ≥1 mm	PA remained unchanged, as well as consumption of vegetables, processed foods, and soy sauce intake. Soup intake reduced significantly at 6 months (authors suggested patients tried to reduce their salt intake)	6 months

BL, baseline; BMI, Body Mass Index; cIMT, carotid Intima-Media Thickness; CT, Computed Tomography; CAC, Coronary Artery Calcification; CHD, Coronary Heart Disease; CAD, Coronary Artery Disease; CUS, Carotid Ultrasound; CVD, Cardiovascular Disease; EBT, Electron Beam Tomography; ICM, Intensive Case Management; PA, Physical Activity; RCT, Randomized Control Trial;

* Research letter;

# Methods have been previously described (35).

**Table 3:** Summary of RCTs on provision of CAC and CUS results and changes in diet, physical activity and anthropometric measures.

**Author, year, country**	**Sample details (size, age, sex, type)**	**Methods and intervention**	**CAC classification**	**Outcomes**	**Findings based on the outcomes of interest**	**Follow-up period**
**Coronary artery calcification (CAC)**						
O'Malley et al. (36), US	Four hundred and fifty active-duty US Army personnel, 79% men, 42 ± 1.9 y, booked for a periodic Army mandated physical examination	Participants were randomly allocated to 1 of 4 interventions: EBT plus either ICM or usual care; EBT not provided plus either ICM or usual care; EBT results included an illustrative picture of the coronary artery with focus on any abnormalities and the CAC score and were provided by the research nurse. Counseling involved risk factor identification and advice in those with CAC, including risk factor management (hypertension, hypercholesterolemia, obesity, high-fat diet, sedentary lifestyle, smoking and glucose intolerance). Participants with risk factors were referred to their physician/dietitian	CAC assessed by EBT, with scores determined according to Agatston	Primary outcome was change in FRS. Secondary outcomes included dietary fat intake, PA and BMI	EBT information, with or without ICM, was not associated with changes in BMI, PA, and dietary fat consumption	12 months
Lederman et al. (37), US	Fifty six post-menopausal women, 64.7 ± 6.9 y, self-referred from press advertisements or were referred by their physician	Participants were randomly allocated to conventional screening or conventional screening plus DHCT. CAC group received images of their CAC and an interpretation from a radiologist. A physician provided a tailored counseling session on nutrition, supplement use, PA, weight control, smoking cessation, and use of HRT and medication for CHD risk reduction to all participants	CAC assessed by DHCT, with scores determined according to Agatston: <10 = very low risk, ≥10- <100 = low risk; ≥100- <400 = medium risk, and ≥400 = high risk for CAD	Primary outcomes included BMI. Other measures included change in dietary fat, fiber, and PA	There was no change in BMI, fat and fiber intake in both the conventional screening group and CT imaging group	6 and 12 months
Rozanski et al. (10), US	Two thousand and one hundred and thirty seven adults with CVD risk factors, 52.5% men, 58.5 ± 8.4 y, from Cedars-Sinai Medical Center	Participants were randomly allocated to no CAC scan or CAC scan. CAC images, score and percentile scores were discussed with those in the CAC group. Nurses provided risk factor counseling session to participants. This comprised provision of a printed customized risk factor management pack with information from the American Heart Association guidelines on cardiac risk factors, results for each risk factor and information on how to improve their risk factors	CAC assessed by EBT, with scores determined according to Agatston	Primary outcome was change in CAD risk factors and FRS. Other measures included weight, WC and PA	WC was significantly lower in those with increased abdominal girth in the scan group, compared with the no-scan group. Weight remained unchanged. Those with higher CAC reported increased exercise (non-exercisers at BL)	4 years
Venkataraman et al. (38)^*^	Four hundred and fifty five adults, 43% men; 59 ± 8 y, with CVD risk and family history of premature CAD from the CAUGHT-CAD trial	Participants underwent standard risk factor management. The CAC-guided arm started on statins	CAC assessed by EBT, with scores determined according to Agatston	Primary outcome was change in anthropometric parameters (weight)	Weight remained the same in both within and between CAC groups	12 months
**Carotid ultrasound (CUS)**						
Rodondi et al. (39), Switzerland	Five hundred and thirty six smokers (≥10 cigarettes/d), 55% men, 51.1 ± 7.3 y, from the general population in the French speaking part of Switzerland self-referred from press advertisements	Participants were randomly allocated to screening or no screening of carotid plaque. Smokers with plaque received 2 images of their plaques and a 7-min tutorial about atherosclerotic plaques. Smokers without plaques and the control group were provided with a 7-min tutorial on the smoking related-risks. All participants underwent a smoking cessation program for 1 year. Information on D&L not reported	Carotid plaques were defined as a focal widening of at least 50% relative to an adjacent segment	Primary outcome was smoking cessation. Other measures included PA	PA did not change in both groups	12 months
Näslund et al. (40), Sweden	Three thousand and five hundred and thirty two adults with ≥1 risk factor, 47% men, 40–60 y, from the Västerbotten Intervention Programme (VIP)	Participants were randomly allocated to receive illustration of CUS plus a nurse phone call or not informed group. Presence of plaque was shown as a traffic light for each carotid artery, with a red circle for a plaque or a green circle for no plaque, and image was included. Written material was provided and contained information about atherosclerosis being a process that is modifiable by a healthy lifestyle and medication. Nurses and physicians provided interpretation of the results and advice on prevention of CVD	Carotid plaque assessed by CUS with cIMT measured in the left and right common carotid arteries	Primary outcomes were FRS and SCORE. Secondary outcomes included weight and WC	There was a slight non-significant decrease in weight in the intervention group. WC remained unchanged	12 months
Bengtsson et al. (12), Sweden	Three thousand and five hundred and thirty two healthy adults aged 40–60 y from the Västerbotten Intervention Programme (VIP)	Participants were randomly allocated to receive CUS pictorial information plus additional information or no pictorial information. CUS results were mailed to the intervention group in a pictorial format. Presence of plaque was illustrated with a red circle while no plaque was a green circle, with cIMT illustrated with a color gage indicating vascular age and ranging from green to yellow, orange and red. Written information on atherosclerosis was provided, with participants receiving healthy lifestyle changes advice to prevent atherosclerosis progression	Carotid plaque assessed by CUS	Primary outcomes were FRS and SCORE. Secondary outcomes included weight and WC	WC was significantly lower in the intervention group. Weight remained unchanged	3 years

BL, baseline; BMI, Body Mass Index; CAC, Coronary Artery Calcification; CAD, Coronary Artery Disease; CHD, Coronary Heart Disease; cIMT, carotid Intima-Media Thickness; CT, Computed Tomography; CUS, Carotid Ultrasound; CVD, Cardiovascular Disease; DHCT, Double-Helical Computed Tomography; EBT, Electron Beam Tomography; FRS, Framingham Risk Score; HRT, Hormone Replacement Therapy; ICM, Intensive Case Management; NRT, Nicotine Replacement Therapy; PA, Physical Activity; RCT, Randomized Control Trial; WC, Waist Circumference; SCORE, Systematic COronary Risk Evaluation;

* Poster.

## Results

### Synthesis of results

Twenty studies (10, 12, 22–34, 36–40) met the inclusion criteria by providing vascular images to motivate changes toward improving diet, physical activity or anthropometric measures. Of these, thirteen were observational studies (22–34) and 7 were RCTs (10, 12, 36–40). Nineteen articles were published within 2006 to 2021, while one study was published in 1996 (Tables 2, 3).

The outcomes of interest found in the eligible studies comprised: overall diet composition (23, 25–27, 29, 31, 34), vegetable intake (34), fiber intake (32, 37), fat and cholesterol intake (22, 24, 36, 37), salt intake (24, 32), consumption of processed foods (34), and amount of physical activity or exercise (10, 22, 23, 25–27, 30–32, 34, 36, 39). These components were chosen due to their role as critical modifiable lifestyle factors to prevent and/or delay the development of CVD. Studies which examined weight and/or BMI (10, 12, 22, 24, 28–30, 32, 33, 36–38, 40) and waist circumference (10, 12, 33, 40) were also included, as those measures are likely to change primarily as a result of changes in diet and/or physical activity. Results regarding the positive and neutral changes on diet, physical activity, and anthropometric measures, due to provision of vascular imaging, for the observational studies and RCTs can be found in Table 4.

**Table 4:** Changes in diet, physical activity and anthropometric measures in observational studies and RCTs.

**Outcomes of interest**	**Observational CAC**	**Observational CUS**	**RCT CAC**	**RCT CUS**
Overall diet	✓✓✓✓✓ (23, 25–27, 29)	xx (31, 34)		
Vegetable intake		x (34)		
Intake of processed foods		x (34)		
Fiber intake		✓(32)	x (37)	
Fat and cholesterol intake	✓✓ (22, 24)		xx (36, 37)	
Salt intake	✓ (24)	✓(32)		
Physical activity/exercise	✓✓✓ (25–27); xx (22, 23)	xxxx (30–32, 34)	x (36);✓(10)	x (39)
Weight/BMI	✓✓✓ (22, 28, 29); x (24)	xxx (30, 32, 33)	xxxx (10, 36–38)	xx (12, 40)
Waist circumference		x (33)	✓ (10)	x (40);✓(12)

BMI, Body Mass Index; CAC, Coronary Artery Calcification; CUS, Carotid Ultrasound; RCT, Randomized Control trial. ✓, significant beneficial change; x, no significant change.

The mean age of participants across studies ranged from 45 to 69 years, and the sample sizes varied from 30 to 2,608. The changes in diet, physical activity or anthropometric measures were investigated over a period of 1 month to 6 years following provision of results. For clarity, the characteristics and findings of the studies are presented separately for observational studies and RCTs, due to the particular characteristics of each type of study.

### Observational studies

#### Participant details and enrolment methods

In the observational studies, participants were asymptomatic (Table 2) and included healthy individuals (22–26, 28, 29), smokers (30), adults with at least one CVD risk factor (27, 31, 32), individuals with hypertension (33) and individuals with type 2 diabetes (34). Participants were recruited by contacting the study investigators directly (22, 30), or referred by physicians (23, 25, 31), or a combination of the above (26–28), and were enrolled at routine clinic visits (29, 32, 33), or at hospitals (34). In one study, investigators recruited a subsample of most residents (82%) from a southern California community (24) (Table 2).

#### Provision of results and diet and lifestyle recommendations

In the observational studies, CAC and CUS results were provided to participants either as images only (23, 25, 28, 32), a combination of images/graphs and/or scores/text (24, 26), pictures followed by an educational tutorial (30), or via a phone call followed by mailed written information (27). Five studies did not report or specify this information (22, 29, 31, 33, 34). The results were provided to participants by physicians (25, 26, 28), a primary care provider (27, 32), mailed to participants (24) or were not reported / not specified (22–24, 29–31, 33, 34). Two observational studies (26, 28) clearly stated that participants were provided with diet and physical activity recommendations, with all other studies mentioning general lifestyle modifications or not reporting on providing any information. Only 38% (*n* = 5) of the studies provided participants with specific recommendations for lifestyle change (25, 26, 28, 30, 32), with one proving specific lifestyle advice to a subgroup with abnormal lipid or glucose levels (30).

#### Duration of follow-up methods

In the observational studies, diet and physical activity changes made by participants were followed up via mail (22, 24, 26, 27, 32), electronically (29) and face-to-face during clinic visits (28, 30, 34). In two studies, follow-up data were obtained via surveys/interviews (25, 31), but the authors did not specify whether surveys were mailed to participants, or whether interviews took place face-to-face or via phone.

#### Qualitative and quantitative outcomes

Results relating to the impact of provision of vascular imaging results on diet, physical activity and anthropometric measures were charted (Table 2). Observational studies exploring the associations between provision of coronary artery and carotid calcification and changes in diet yielded mixed results. Five studies (out of seven studies) observed a positive change in overall diet measures (23, 25–27, 29). Improvements in dietary fiber (32), fat (22, 24) and salt (24, 32) were observed. However, no changes were observed for consumption of vegetables (34) or processed foods (34). Positive changes in weight and/or BMI were observed in three studies (out of seven studies) (22, 28, 29). However, waist circumference remained similar (33). Three studies (25–27) reported an increase in physical activity levels after provision of vascular imaging results (Table 2).

### Randomized controlled trials

#### Participant details and enrolment methods

In the RCTs (Table 3), participant were either smokers (39), post-menopausal women (37), adults with CVD risk factors (10, 38, 40), and otherwise healthy individuals (12, 36). Sample sizes varied from 56 to 3,532 and the mean/median age of participants ranged from 42 to 65 years. The duration of interventions in the RCTs ranged from 6 months to 4 years. Across RCTs, participants were recruited from previous trials (12, 38, 40), a medical center (10), self-referral (39), both self-referral or referral from physicians (37), or a mandatory periodic physical examination (36) (Table 3).

#### Provision of results and diet and lifestyle recommendations

In the RCTs, CAC and CUS results were mainly provided as images (36), or a combination of images with written information (12, 37, 40), including scores (10), as well as a video (39). In one of the studies this information was not reported (38). Except for two RCTs that reported that the results were delivered to participants by a research nurse (10, 36) and via mail (12), this information in all others studies was not specifically stated (37–40). Only two RCTs (36, 37) clearly specified that counseling on diet and physical activity was provided to participants. All other RCTs (*n* = 5) provided counseling with focus on risk factor management and lifestyle without explicitly mentioning diet and physical activity (10, 12, 38–40). Further details have been reported in Table 3.

#### Follow-up methods

RCT study participants were followed-up at clinic visits after 6 months to 4 years following provision of results (10, 12, 36–40).

#### Qualitative and quantitative outcomes

The RCTs focused on changing Framingham Risk Score (FRS) (10, 12, 36, 40), Systematic Coronary Risk Evaluation (SCORE) (12, 40), smoking cessation (39), and improving risk factors such as blood pressure and lipids, rather than diet, physical activity and anthropometric measures. Therefore, these trials were not specifically designed to examine whether provision of CAC and CUS could lead to healthy changes in diet, physical activity and anthropometric measures. Only two RCTs (37, 38) included BMI and body weight within their primary outcomes. In all other RCTs, diet, physical activity and anthropometric measures were considered secondary or non-specified outcomes (Table 3).

#### CAC/CUS scan vs. no scan

Findings from seven RCTs included in this scoping review (Table 5) showed that provision of scan results did not lead to changes in the intake of dietary fiber and fat a year later, compared to the no scan group (37). Similarly, BMI remained unchanged in both the scan and no scan groups (37). In the EISNER (Early Identification of Subclinical Atherosclerosis by non-invasive Imaging Research) study (10), when comparing the CAC scan group with the non-CAC scan group, investigators observed a significant decrease in waist circumference at 4 years in a subgroup with increased waist circumference (M>40, W>35) at baseline (Table 5), whereas body weight remained unchanged (10). Provision of vascular imaging did not lead to changes in physical activity levels in the scan group, compared to no scan group over 1 year (39) and 4 years (10).

**Table 5:** Changes in diet, physical activity and anthropometric measures according to CAC and CUS scores in RCTs.

**Randomized controlled trials - CAC groups**		**Change**	***P*** **-value**
**O'Malley et al. (** **36** **), US**		**12 months**	
Change in BMI, kg/m2, mean (SE)	CAC informed vs. CAC not informed	0.38 (0.12)	0.84
Change in exercise^a^, sports index, mean (SE)	CAC informed vs. CAC not informed	0.02 (0.05)	0.23
**Lederman et al. (** **37** **), US**		**12 months**	
Change in BMI, kg/m2, mean (SD)	CAC plus Counseling vs. Counseling alone (no CAC scan)	0.10 (1.33)	>0.05
Change in fiber intake, *n* (%)	CAC plus Counseling vs. Counseling alone (no CAC scan)	3 (13.0%)	>0.05
Change in fat intake, *n* (%)	CAC plus Counseling vs. Counseling alone (no CAC scan)	7 (29.2%)	>0.05
**Rozanski Al et al. (** **10** **), US**		**4 years**	
Change in weight, kg, median (IQR)	CAC scan vs. no CAC scan (BM I≥25 kg/m2 at baseline)	0 (−2.72, 3.17)	0.07
Change in WC, cm, median (IQR)	CAC scan vs. no CAC scan (increased WC [M>40, W>35] at baseline)	0 (−7.62, 5.08)	**0.01**
Change in exercise, ≥3 times/week, *n* (%)	CAC scan vs. no CAC scan (no exercise at baseline)	214/582 (37%)	0.77
Change in weight, kg, median (IQR)	Highest vs. lowest CAC scores (BMI ≥25 kg/m2 at baseline)	−3 (−10, 3)	**<0.001**
Change in WC, cm, median (IQR)	Highest vs. lowest CAC scores (increased WC [M>40, W>35] at baseline)	−1 (3.3, 0.5)	0.56
Change in exercise, ≥3 times/week, *n* (%)	Highest vs. lowest CAC scores (no exercise at baseline)	17/36 (47%)	**0.03**
**Venkataraman et al. AU** **(****38****)**		**12 months**	
Change in weight, kg, mean	CAC informed vs. CAC not informed	3.92	0.30
Change in weight, kg, mean	Highest vs. lowest CAC scores	−0.12	0.11
**Randomized controlled trials - CUS groups**			
**Rodondi et al. (** **39** **), Switzerland**		**12 months**	
Change in exercise, MET min/wk, mean (SE)	CUS scan vs. no CUS scan	−784 (205)	0.98
**Näslund et al. (** **40** **), Sweden**		**12 months**	
Change in weight, Kg, mean (95%CI)	CUS informed plus a nurse phone call vs. CUS not informed	1.62 (−0.06, 3.30)	>0.05
Change in WC, cm, mean (95%CI)	CUS informed plus a nurse phone call vs. CUS not informed	1.3 (−0.01, 2.60)	>0.05
**Bengtsson et al. (** **12** **), Sweden**		**3 years**	
Change in weight, Kg, mean (95%CI)	CUS informed vs. CUS not informed	0.8227 (−0.2186, 1.864)	>0.05
Change in WC, cm, mean (95%CI)	CUS informed vs. CUS not informed	0.8681 (0.0072, 1.729)	**0.032**

BMI, Body Mass Index; CAC, Coronary Artery Calcification; CUS, Carotid Ultrasound, WC, Waist Circumference.

a Baecke Physical activity questionnaire – sports index ranges from 0 to 5. *P*-values marked in bold indicate statistically significant results.

#### CAC/CUS scan informed vs. not informed

Compared to participants who were not informed of their scan results, provision of vascular imaging did not lead to reductions in body weight (12, 38, 40) or BMI (36). Waist circumference did not change after 1 year in one study (40), but provision of CUS led to significant reductions in another study (12) after 3 years of provision of results, compared to the group not informed (Table 5). No changes in physical activity were observed in participants after a year of being informed of their vascular results, compared to those who were not informed (36).

#### Evidence vs. no evidence of CAC/CUS (zero/low to higher scores)

When examining the changes within groups according to CAC/CUS scores in the EISNER study (10), participants who were overweight and had a CAC score >100 had a greater weight loss after 4 years (Table 5). However, no significant changes in body weight were observed within groups in the Venkataraman trial (38). Participants with higher CAC scores in the EISNER study (10), who were non-exercisers at baseline, reported an increase in exercise levels after 4 years.

### Clinical assessments and reporting in both observational and RCT studies

A range of instruments were used to assess diet and physical activity in the eligible studies, with many being self-reported or single-item questions (Supplementary Tables S1, S2, for observational and RCT studies, respectively). The results were also reported in several different ways (i.e., mean ± SE, mean (SE), median and interquartile range, mean and 95% confidence interval) and given in dissimilar unit measures (i.e., MET, min/week, percentages), which makes comparability of results difficult.

## Discussion

In this scoping review, we identified many gaps and opportunities to inform the design of future high quality RCTs providing vascular imagining results to elicit positive changes to diet and physical activity. We revealed three key messages. First, the results of observational studies gathered in this scoping review suggest that providing CAC and CUS imaging could lead to healthy changes to diet, physical activity, and anthropometric measures (weight, BMI and waist circumference). However, well-designed clinical trials with particular focus on improving diet and physical activity and reducing anthropometric measures (rather than focused on CVD risk factor management) are needed to strengthen quality of evidence regarding the impact of CAC and CUS imaging results on these particular lifestyle behaviors. Secondly, the outcomes of interest in the present review were mainly secondary or non-specified outcomes for the studies included. These studies were largely designed to answer a broader question, focusing mainly on estimated cardiovascular risk or smoking cessation. Finally, the methodologies used varied significantly. For instance, few studies included counseling sessions or other well-recognized behavior change techniques, such as methods for self-monitoring (41). Studies using provision of vascular health imagining eliciting changes on CVD risk have demonstrated mixed results. There is need for interventions to be tested in large clinical trials before implementing in clinical practice (15).

### Overall results

Twenty studies examining the impact of provision of CAC/CUS imaging results on diet and/or physical activity were reviewed. Of these, twelve studies focused on CAC (four RCTs and eight observational) and eight studies focused on CUS (three RCTs and five observational). Half of the participants were free of CVD and the other half were at high risk for CVD (at least 1 CVD risk factor, i.e., smokers, hypertensive). Most studies reported providing written/verbal/imaging results but only some studies clearly stated they provided dietary and physical activity advice when providing vascular imaging results. None of the RCTs had dietary or physical activity as a primary outcomes and assessment and reporting of the outcomes were suboptimal and inconsistent among studies. The considerations discussed below can potentially help with the design of future studies where participants receive appropriate guidance and support after provision of vascular imaging, which can be translated into significant changes to their diet and physical activity.

### Diet and physical activity as non-primary outcomes

We observed that the primary outcomes of most of the studies included in this scoping review focused on managing blood pressure and smoking cessation, rather than changing diet, physical activity and anthropometric measures. Hence, the recommendations for behavior changes were focused on medication and other therapies specific to manage those outcomes. Although some RCTs provided information on risk factor management (10, 36, 38, 39), specific recommendations to promote a healthy diet and lifestyle, beyond risk factor management, was a missed opportunity. Evidence shows that providing this information grounded in theory can produce change, helping people to make healthier choices. Such changes have the potential to translate to a more successful change in diet, physical activity and body weight as well as risk factor management.

### Inconsistent methodologies among studies

In general, the methodologies of the studies included in this review varied greatly, making it difficult to determine which study design would have been potentially more promising at leading to positive changes in diet and physical activity. These included: disparities in randomization methods (i.e., CAC informed group vs. not informed, or CAC performed vs. not performed), instruments used to assess diet and physical activity (e.g., no reference to questionnaires being validated), how the messages were conveyed (including a lack of clarity in how this was performed), which recommendations for lifestyle change were provided (i.e., recommendations from national/international guidelines), and by whom (i.e., clinician, counselor, researcher, etc.). Some observational studies reported physicians had delivered the results to participants, although in most of the observational studies, this was not specified. Only one RCT clearly stated that a physician provided the participants with their results; in all others, this information was not reported or not specified. Previous studies have shown that interventions are more likely to be unsuccessful if not delivered by a clinician (36). In addition, individuals are unlikely to make changes to their diet and lifestyle if no guidance is provided on how to achieve specific goals to improve risk factors (36).

Overall, behavior change has been shown to be possible but needs to be deliberately included and designed, taking advantage of the “teachable moment” (42). These are brief moments during life when people are more receptive to behavior change messages, and these moments can be used to encourage individuals to change unhealthy behaviors (43). The findings of this study indicate that future well-designed interventions should not only focus on providing recommendations on diet and physical activity change, but equally important, follow up, motivate, and support individuals, to enhance the likelihood of achieving significant beneficial changes.

The use of behavior change techniques (BCTs) appears to positively impact the effectiveness of behavior change interventions. A meta-analysis investigating e-health interventions to increase fruit and vegetable intake observed that interventions using 7–8 BCTs (*n* = 4) were more successful compared with interventions using six or less BCTs (44). The following 5 BCTs identified as more commonly used in studies appeared to equally positively influence the study interventions (44): i. *Provide directions on how to change behavior; ii. Provide feedback on performance; iii. Identification of barriers; iv. Goal Setting* and v. *Inform on consequences of behaviors* (44). This suggests that adding BCT may improve effectiveness of interventions, by providing further support to individuals to achieve and maintain a healthy lifestyle (37).

Evidence shows that presence of CAC, rather than simply having a scan performed, leads to a greater motivation toward improving CVD risk factors (19). Moreover, participants with presence of calcification, and more importantly those with higher risk factors for CVD (14) seem to be more motivated to change their behavior (37), with interventions being more successful (14) among those with higher risk. Conversely, having a normal vascular image (no calcification) has been suggested to discourage behavior change toward risk factors (37), but the evidence for this remains limited. This highlights the importance of clarifying to individuals that changes in diet and physical activity, do not only slow the progression of CVD, but can also prevent the onset of vascular-related conditions, particularly in those with CVD risk factors.

Furthermore, vascular imaging has been shown to improve CVD management without leading to great increase in medical costs to participants (10). The need for larger clinical trials has been suggested to confirm whether these findings can be extended to more diverse populations, as well as to investigate whether provision of vascular imaging and the subsequent improvement in CVD risk factors lead to a lower rate of cardiovascular events (10).

A systematic review and meta-analysis including 21 RCTs published in 2022 (45) reported the potential of provision of medical images to encourage risk-reducing behaviors and reduce risk factors. However, they indicated the need for further satisfactorily powered trials well-controlled for risk of bias (45). The review also suggested that building further evidence for some key behavioral outcomes is warranted, as most trials focused on medication use and adherence, smoking cessation, and increase physical activity (45). A study protocol investigating whether provision of vascular imagining to individuals could significantly lead to beneficial behavior change, including diet and physical activity among other behavior change outcomes has been previously published (46) with the RCT underway. Results of this study may contribute to further understanding and potentially strengthening the current evidence that provision of imagining can encourage health behavior modification.

Overall, the data summarized in this review contributes to the literature on the topic and can serve as a reference for future clinical trials and as a guide for future research. We have identified numerous opportunities to improve interventions aiming to promote changes to diet and physical activity via the provision of vascular imaging.

### Limitations

A limitation was the lack of studies specifically designed to answer the question on whether visualization of non-invasive vascular imaging results could lead to beneficial changes in diet and physical activity, and as such it is difficult to evaluate specific knowledge gaps or beneficial approaches. Most of the existing studies focused on smoking cessation and improving estimated cardiovascular risks, with changes in diet and physical activity being mainly secondary outcomes. In addition, the outcomes of interest were assessed using several different instruments. Noteworthy, physical activity/exercise levels were estimated using different and non-comparable instruments (i.e., questions to estimate physical activity [yes/no questions], and the disparities in reporting the results [i.e., MET min/week, mean ± SE]) made comparison difficult. Furthermore, the terms exercise and physical activity were used interchangeably in many studies (i.e., self-reported physical activity was referred as exercise – structured exercise such as going to the gym). As physical activity and dietary intake data was mostly self-reported, these data should be interpreted with caution.

## Conclusion

The results of this scoping review suggest the need for well-structured interventions with the objective of motivating individuals to make positive changes to their diet and physical activity. Although there is consistent evidence that provision of non-invasive vascular imaging results can encourage individuals to make positive behavior changes in relation to medication adherence and other CVD risk factors, evidence for its impact on diet and physical activity remains very weak. However, this is largely due to the lack of studies designed to address this question. Well-designed clinical trials are warranted to further clarify and strengthen the beneficial results mainly seen in observational studies. Future clinical trials should consider a consistent and clear message on how to achieve positive diet, physical activity and other lifestyle changes, preferably be delivered by a trained counselor, and include a follow up session to reinforce advice, ensure understanding, and to provide further encouragement to support positive diet and physical activity changes.

## Data availability statement

The original contributions presented in the study are included in the article/Supplementary material, further inquiries can be directed to the corresponding author/s.

## Author contributions

SR-B searched the literature and retrieved the articles. SR-B and AKG screened the papers for eligibility. SR-B, MAK, and JRL participated in the writing of the manuscript. SR-B, AKG, MAK, MS, LCB, CPB, BJ, JD, MPS, JMH, and JRL contributed to the work's conception and design, data interpretation, critical revision of the manuscript, and approved the version of the manuscript being submitted. All authors contributed to the article and approved the submitted version.

## Funding

This study received no specific grant from any funding agency in the public, commercial or not-for-profit sectors. The salary of MS was supported by a Royal Perth Hospital Research Foundation Career Advancement Fellowship (ID: CAF 130/2020) and an Emerging Leader Fellowship from the Western Australian Future Health Research and Innovation Fund. The salary of LCB was supported by a National Health and Medical Research Council (NHMRC) of Australia Emerging Leadership Investigator Grant (ID: 1172987) and a National Heart Foundation of Australia Post-Doctoral Research Fellowship (ID: 102498). The salary of CPB was supported by a Royal Perth Hospital Research Foundation Lawrie Beilin Career Advancement Fellowship (ID: CAF 127/2020). The salary of JMH was supported by a National Health and Medical Research Council of Australia Senior Research Fellowship (ID: 1116973). The salary of JRL was supported by a National Heart Foundation of Australia Future Leader Fellowship (ID: 102817). None of the funding agencies had any role in the conduct and management of the study, data interpretation, preparation, review, or approval of the manuscript.

## Conflict of interest

The authors declare that the research was conducted in the absence of any commercial or financial relationships that could be construed as a potential conflict of interest.

## Publisher's note

All claims expressed in this article are solely those of the authors and do not necessarily represent those of their affiliated organizations, or those of the publisher, the editors and the reviewers. Any product that may be evaluated in this article, or claim that may be made by its manufacturer, is not guaranteed or endorsed by the publisher.

## References

[1] WHO. Cardiovascular diseases (CVDs). World Health Organization (2021). Available online at: https://www.who.int/news-room/fact-sheets/detail/cardiovascular-diseases-(cvds) (accessed October 6, 2022).

[2] McClellan M Brown N Califf RM Warner JJ. Call to action: urgent challenges in cardiovascular disease: a presidential advisory from the American heart association. Circulation. (2019) 139:e44–54. 10.1161/CIR.0000000000000652 30674212

[3] Levine DA Davydow DS Hough CL Langa KM Rogers MA Iwashyna TJ. Functional disability and cognitive impairment after hospitalization for myocardial infarction and stroke. Circ Cardiovascu Qual Outcom. (2014) 7:863–71. 10.1161/HCQ.0000000000000008 25387772 PMC4241126

[4] Ezzati M Riboli E. Behavioral and dietary risk factors for noncommunicable diseases. N Eng J Med. (2013) 369:954–64. 10.1056/NEJMra1203528 24004122

[5] Artinian NT Fletcher GF Mozaffarian D Kris-Etherton P Van Horn L Lichtenstein AH . Interventions to promote physical activity and dietary lifestyle changes for cardiovascular risk factor reduction in adults. A scientific statement from the American heart association. Circulation. (2010) 122:406–41. 10.1161/CIR.0b013e3181e8edf1 20625115 PMC6893884

[6] Wang X Ouyang Y Liu J Zhu M Zhao G Bao W . Fruit and vegetable consumption and mortality from all causes, cardiovascular disease, and cancer: systematic review and dose-response meta-analysis of prospective cohort studies. Bmj. (2014) 349:g4490. 10.1136/bmj.g4490 25073782 PMC4115152

[7] Lee-Kwan SH Moore LV Blanck HM Harris DM Galuska D. Disparities in state-specific adult fruit and vegetable consumption—United States, 2015. MMWR Morbid Mortal Week Rep. (2017) 66:1241. 10.15585/mmwr.mm6645a1 29145355 PMC5726245

[8] ABS. Australian Health Survey: Consumption of food groups from the Australian Dietary Guidelines, Australia 2011–12. Australian Bureau of Statistics (2016).

[9] ABS. Physical activity: *Contains Key Statistics And Information About Exercise and Physical Activity Trends Within Australia, Including State and Territory Specific Findings*. Australian Bureau of Statistics (2018).

[10] Rozanski A Gransar H Shaw LJ Kim J Miranda-Peats L Wong ND . Impact of coronary artery calcium scanning on coronary risk factors and downstream testing the EISNER (early identification of subclinical atherosclerosis by noninvasive imaging research) prospective randomized trial. J Am Coll Cardiol. (2011) 57:1622–32. 10.1016/j.jacc.2011.01.019 21439754 PMC3104928

[11] Mamudu HM Paul TK Veeranki SP Budoff M. The effects of coronary artery calcium screening on behavioral modification, risk perception, and medication adherence among asymptomatic adults: a systematic review. Atherosclerosis. (2014) 236:338–50. 10.1016/j.atherosclerosis.2014.07.022 25128971

[12] Bengtsson A Norberg M Ng N Carlberg B Grönlund C Hultdin J . The beneficial effect over 3 years by pictorial information to patients and their physician about subclinical atherosclerosis and cardiovascular risk: Results from the VIPVIZA randomized clinical trial. Am J Prevent Cardiol. (2021) 7:100199. 10.1016/j.ajpc.2021.100199 34611639 PMC8387279

[13] Glanz K Rimer BK. Theory at a glance: A guide for health promotion practice. National Cancer Institute, NIH. Public Health Service. Washington, DC: US Government Printing Office (2005).

[14] Greenland P. Improving risk of coronary heart disease: can a picture make the difference? Jama. (2003) 289:2270–2. 10.1001/jama.289.17.2270 12734139

[15] Rodondi N Auer R de Bosset Sulzer V Ghali WA Cornuz J. Atherosclerosis screening by noninvasive imaging for cardiovascular prevention: a systematic review. J Gen Intern Med. (2012) 27:220–31. 10.1007/s11606-011-1833-3 21882076 PMC3270245

[16] Joshi PH Patel B Blaha MJ Berry JD Blankstein R Budoff MJ . Coronary artery calcium predicts cardiovascular events in participants with a low lifetime risk of cardiovascular disease: the Multi-Ethnic Study of Atherosclerosis (MESA). Atherosclerosis. (2016) 246:367–73. 10.1016/j.atherosclerosis.2016.01.017 26841074

[17] Finn AV Kolodgie FD Virmani R. Correlation between carotid intimal/medial thickness and atherosclerosis: a point of view from pathology. Arterioscler Thromb Vasc Biol. (2010) 30:177–81. 10.1161/ATVBAHA.108.173609 19679833

[18] Yeboah J McClelland RL Polonsky TS Burke GL Sibley CT O'Leary D . Comparison of novel risk markers for improvement in cardiovascular risk assessment in intermediate-risk individuals. Jama. (2012) 308:788–95. 10.1001/jama.2012.9624 22910756 PMC4141475

[19] Gupta A Lau E Varshney R Hulten EA Cheezum M Bittencourt MS . The identification of calcified coronary plaque is associated with initiation and continuation of pharmacological and lifestyle preventive therapies: a systematic review and meta-analysis. JACC: Cardiovasc Imag. (2017) 10:833–42. 10.1016/j.jcmg.2017.01.030 28797402 PMC5761651

[20] Arksey H O'Malley L. Scoping studies: towards a methodological framework. Int J Soc Res Methodol. (2005) 8:19–32. 10.1080/1364557032000119616

[21] Tricco AC Lillie E Zarin W O'Brien KK Colquhoun H Levac D . PRISMA extension for scoping reviews (PRISMA-ScR): checklist and explanation. Ann Intern Med. (2018) 169:467–73. 10.7326/M18-0850 30178033

[22] Wong ND Detrano RC Diamond G Rezayat C Mahmoudi R Chong EC . Does coronary artery screening by electron beam computed tomography motivate potentially beneficial lifestyle behaviors? Am J Cardiol. (1996) 78:1220–3. 10.1016/S0002-9149(96)00599-1 8960578

[23] Kalia NK Miller LG Nasir K Blumenthal RS Agrawal N Budoff MJ. Visualizing coronary calcium is associated with improvements in adherence to statin therapy. Atherosclerosis. (2006) 185:394–9. 10.1016/j.atherosclerosis.2005.06.018 16051253

[24] Sandwell JC Wingard DL Laughlin GA Barrett-Connor E. Electron beam computed tomography screening and heart disease risk factor modification. Prev Cardiol. (2006) 9:133–7. 10.1111/j.1520-037X.2006.04862.x 16849875

[25] Orakzai RH Nasir K Orakzai SH Kalia N Gopal A Musunuru K . Effect of patient visualization of coronary calcium by electron beam computed tomography on changes in beneficial lifestyle behaviors. Am J Cardiol. (2008) 101:999–1002. 10.1016/j.amjcard.2007.11.059 18359321

[26] Schwartz J Allison M Wright CM. Health behavior modification after electron beam computed tomography and physician consultation. J Behav Med. (2011) 34:148–55. 10.1007/s10865-010-9294-4 20857186 PMC3048462

[27] Johnson JE Gulanick M Penckofer S Kouba J. Does knowledge of coronary artery calcium affect cardiovascular risk perception, likelihood of taking action, and health-promoting behavior change? J Cardiovasc Nursi. (2015) 30:15–25. 10.1097/JCN.0000000000000103 24434820

[28] Kalia NK Cespedes L Youssef G Li D Budoff MJ. Motivational effects of coronary artery calcium scores on statin adherence and weight loss. Coron Artery Dis. (2015) 26:225–30. 10.1097/MCA.0000000000000207 25514570

[29] Schurink M Braber T Prakken N Doevendans P Backx F Grobbee D . No psychological distress in sportsmen aged 45 years and older after cardiovascular screening, including cardiac CT: the Measuring Athlete's Risk of Cardiovascular events (MARC) study. Netherlands Heart Journal. (2017) 25:271–7. 10.1007/s12471-017-0948-5 28144819 PMC5355386

[30] Rodondi N Auer R Devine PJ O'Malley PG Hayoz D Cornuz J. The impact of carotid plaque screening on motivation for smoking cessation. Nicotine and tobacco research. J Soc Res Nicotine Tobacco. (2008) 10:541–6. 10.1080/14622200801902011 18324574

[31] Johnson HM Einerson J Korcarz CE Aeschlimann SE Stein JH. Long-term effects of carotid screening on patient outcomes and behaviors. Arch Intern Med. (2011) 171:589–91. 10.1001/archinternmed.2011.90 21444853 PMC3075090

[32] Johnson HM Turke TL Grossklaus M Dall T Carimi S Koenig LM . Effects of an office-based carotid ultrasound screening intervention. J Am Soc Echocardio. (2011) 24:738–47. 10.1016/j.echo.2011.02.013 21477989 PMC3279118

[33] Hong S-J Chang H-J Song K Hong G-R Park SW Kang H-J . Impact of atherosclerosis detection by carotid ultrasound on physician behavior and risk-factor management in asymptomatic hypertensive subjects. Clin Cardiol. (2014) 37:91–6. 10.1002/clc.22220 24193449 PMC6649369

[34] Jeong I-K Kim S-G Cho DH Kim CH Kim CS Lee W-Y . Impact of carotid atherosclerosis detection on physician and patient behavior in the management of type 2 diabetes mellitus: a prospective, observational, multicenter study. BMC Cardiovasc Disord. (2016) 16:220. 10.1186/s12872-016-0401-5 27842497 PMC5109726

[35] Braber TL Mosterd A Prakken NH Rienks R Nathoe HM Mali WP . Occult coronary artery disease in middle-aged sportsmen with a low cardiovascular risk score: the measuring athlete's risk of cardiovascular events (MARC) study. Eur J Prev Cardiol. (2016) 23:1677–84. 10.1177/2047487316651825 27222386

[36] O'Malley PG Feuerstein IM Taylor AJ. Impact of electron beam tomography, with or without case management, on motivation, behavioral change, and cardiovascular risk profile: a randomized controlled trial. JAMA. (2003) 289:2215–23. 10.1001/jama.289.17.2215 12734132

[37] Lederman J Ballard J Njike VY Margolies L Katz DL. Information given to postmenopausal women on coronary computed tomography may influence cardiac risk reduction efforts. J Clin Epidemiol. (2007) 60:389–96. 10.1016/j.jclinepi.2006.07.010 17346614

[38] Venkataraman P Huynh Q Marwick TH Investigators C-C. Improving cardiovascular prevention through visualisation of CAC: insights from the CAUGHT-CAD trial. J Am Coll Cardiol. (2020) 75:2064. 10.1016/S0735-1097(20)32691-7

[39] Rodondi N Collet T-H Nanchen D Locatelli I Depairon M Aujesky D . Impact of carotid plaque screening on smoking cessation and other cardiovascular risk factors: a randomized controlled trial. Arch Intern Med. (2012) 172:344–52. 10.1001/archinternmed.2011.1326 22269590

[40] Näslund U Ng N Lundgren A Fhärm E Grönlund C Johansson H . Visualization of asymptomatic atherosclerotic disease for optimum cardiovascular prevention (VIPVIZA): a pragmatic, open-label, randomised controlled trial. Lancet. (2019) 393:133–42. 10.1016/S0140-6736(18)32818-6 30522919

[41] Michie S Abraham C Whittington C McAteer J Gupta S. Effective techniques in healthy eating and physical activity interventions: a meta-regression. Health psychology. (2009) 28:690. 10.1037/a0016136 19916637

[42] Lawson PJ Flocke SA. Teachable moments for health behavior change: a concept analysis. Patient Educ Couns. (2009) 76:25–30. 10.1016/j.pec.2008.11.002 19110395 PMC2733160

[43] Flocke SA Clark E Antognoli E Mason MJ Lawson PJ Smith S . Teachable moments for health behavior change and intermediate patient outcomes. Patient Educ Couns. (2014) 96:43–9. 10.1016/j.pec.2014.03.014 24856449 PMC4427843

[44] Rodriguez Rocha NP Kim H. eHealth interventions for fruit and vegetable intake: a meta-analysis of effectiveness. Health Edu Behav. (2019) 46:947–59. 10.1177/1090198119859396 31347403

[45] Hollands GJ Usher-Smith JA Hasan R Alexander F Clarke N Griffin SJ. Visualising health risks with medical imaging for changing recipients' health behaviours and risk factors: Systematic review with meta-analysis. PLoS Med. (2022) 19:e1003920. 10.1371/journal.pmed.1003920 35239659 PMC8893626

[46] Radavelli-Bagatini S Bondonno CP Sim M Blekkenhorst LC Anokye R Connolly E . Modification of diet, exercise and lifestyle (MODEL) study: a randomised controlled trial protocol. BMJ Open. (2020) 10:e036366. 10.1136/bmjopen-2019-036366 33177129 PMC7661361

