# Supplementary material for: Provision of non-invasive coronary and carotid vascular imaging results on changes in diet and physical activity in asymptomatic adults: A scoping review
Source: Front Nutr. 2022 Oct 28;9:946378. doi: 10.3389/fnut.2022.946378 (PMC9650649; doi:10.3389/fnut.2022.946378)
Supplement: Supplementary file 1 [file Data_Sheet_1.docx]

**SUPPLEMENTARY MATERIAL**

**Supplementary table 1.** Instruments used to assess diet, physical activity and anthropometric measures in the observational studies

| **Observational studies - CAC** | |
| --- | --- |
| Wong ND, 1996 (22), US | Questions: have you lost **weight**, decreased **dietary fat**, and increased **exercise** (~1-2y after the initial CAC scan) |
| Kalia NK, 2006 (23), US | Questionnaires about lifestyle modifications including **diet** and **exercise/physical activity** |
| Sandwell JC, 2006 (24), US | Questions on **diet** and/or **exercise** changes after receiving the scan results (6 months after their scan), or whether they already had a “heart healthy” lifestyle before the scan |
| Orakzai RH, 2008 (25), US | Questions on **diet** and **exercise/physical activity** improvements (categorical, yes/no) |
| Schwartz J, 2011 (26), US | Questionnaires about **diet** and **exercise**. Changes 6y later were also assessed by questionnaires using specific questions, including whether participants had initiated exercising, increased **exercise**, changed exercise type, changed their diet, or use of alcohol, since their CAC scan |
| Johnson HM, 2015 (27), US | Open question ‘‘What behavior changes have you made since your scan?” Outcomes of interest: **diet** and **exercise** |
| Kalia NK, 2015 (28), US | Methods for assessing **weight** not specified |
| Schurink, MM, 2017 (29), The Netherlands | Methods for assessing **weight** and **diet** not specified |
| **Observational studies - CUS** | |
| Rodondi N, 2008 (30), Switzerland | **Physical activity** was assessed using the validated International Physical Activity Questionnaire (IPAQ). Body **weight** was measured at the clinic visit (methods not reported) |
| Johnson HM, 2011 (31), US | Questionnaire for assessing **diet** and **exercise** not reported |
| Johnson HM, 2011 (32), US | Self-reported dietary **fiber** and **salt** intake. Participants received an additional survey via mail, and self-reported any behavioral changes, including **exercise** (no further details reported) |
| Hong SJ, 2014 (33), Korea | **Weight** and **waist circumference** were measured at routine office visits |
| Jeong I, 2016 (34), Korea | Questionnaires administered to assess **diet** and **physical activity** using one-on-one interviews and self-reporting techniques |

**Supplementary table 2.** Instruments used to assess diet, physical activity and anthropometric measures in the RCTs

| **Randomized controlled trials - CAC** | |
| --- | --- |
| O'Malley PG, 2003 (36), US | **Physical activity** was assessed using the validated Baecke Physical Activity Questionnaire (sports index ranges from 0 to 5) |
| Lederman J, 2007 (37), US | A health risk assessment including dietary pattern (**fat** and **fiber**) was performed. Healthy Valley 2000. Project CoNECT: health surveys. http://www.healthfulvalley.org/hra3.html |
| Rozanski Al, 2011 (10), US | **Physical activity** was assessed by the question: “Do you exercise regularly (3 to 4 times a week) for at least 30 minutes each time?” (yes/no as response options). **Weight** and **waist circumference** were measured at the clinic visits |
| Venkataraman P, 2020, AU ([38](#_ENREF_37)) | Methods not reported |
| **Randomized controlled trials - CUS** | |
| Rodondi N, 2012 (39), Switzerland | **Physical activity** was assessed using the validated IPAQ |
| Näslund U, 2019 (40), Sweden | **Weight** was measured using a calibrated scale with participants wearing light clothing without shoes. **Waist circumference** was measured using a non-elastic measuring tape, on the skin at the point mid-way between the last palpable rib and the iliac crest |
| Bengtsson A (12), Sweden | Methods for assessing **weight** and **waist circumference** not provided |
